# Head nurses’ transformational leadership and nurses’ job engagement: the mediating role of horizontal violence among nurses

**DOI:** 10.3389/fpubh.2025.1615609

**Published:** 2025-10-10

**Authors:** Xu Yan Liu, Ren Long Liang, Yi Wei Li, Yin Yuan, Tingting Ruan

**Affiliations:** ^1^Department of Gynecology, Deyang People’s Hospital, Deyang, China; ^2^Department of Neurology, Deyang Hospital Affiliated Hospital of Chengdu University of Traditional Chinese Medicine, Deyang, China

**Keywords:** nurses, transformational leadership style, horizontal violence, job engagement, mediation analysis

## Abstract

**Background:**

Horizontal violence among nurses is relatively prevalent and may affect nurses’ level of job engagement. By integrating Affective Events Theory (AET) and Conservation of Resources Theory (COR), This study aims to verify the mediating effect of horizontal violence among nurses on the relationship between head nurses’ transformational leadership style and nurses’ job engagement. It is expected to provide a new perspective for the innovation of theoretical application in the field of nursing management.

**Methods:**

This study adopted a cross-sectional design, A total of 317 nurses from five tertiary grade A general hospitals in Southwest China. Data were collected through a general information questionnaire, a transformational leadership questionnaire, a simplified version of the Utrecht Work Engagement Scale, and an inter-nurse horizontal violence questionnaire. SPSS 26.0 was used for correlation analysis, and Amos 26.0 for constructing the structural equation model, and the Bootstrap method for testing the mediating effect.

**Results:**

The results indicated that horizontal violence among nurses partially mediated the relationship between head nurses’ transformational leadership style and nurses’ job engagement. Specifically, head nurses’ transformational leadership style could not only directly improve nurses’ job engagement but also indirectly enhance it by reducing horizontal violence. Horizontal violence among nurses plays a partial mediating role in the relationship between head nurses’ transformational leadership style and nurses’ job engagement.

**Conclusion:**

Nursing managers should focus on cultivating and selecting head nurses with a transformational leadership style, and implement interventions from two dimensions: regulating affective events and interrupting resource depletion. For instance, by recognizing contributions, supporting innovation, providing care and support, making fair decisions, and establishing effective communication mechanisms, a positive organizational culture can be fostered. This will thereby reduce horizontal violence among nurses and improve their job engagement.

## 1 Introduction

Job engagement refers to a positive psychological state exhibited by employees in their work, characterized by full dedication to work tasks. It can improve nurses’ work efficiency, promote work innovation, enhance nursing quality, and reduce employees’ turnover intention (1). As the primary force in safeguarding public health, nurses play a crucial role. However, in China, the number of registered nurses per 10,000 people is far lower than that in developed countries, making the nurse shortage a particularly prominent issue (2). Excessive workload among nurses has led to a decline in their job engagement, which urgently requires attention (3).

Transformational leadership is one of the mainstream leadership styles currently. Previous studies have shown that transformational leadership exerts various positive effects on employees: among nurses, it can increase job engagement (4), promote innovation (5), and improve nursing quality (6). Nevertheless, the underlying mechanism through which head nurses’ transformational leadership influences nurses’ job engagement remains under-explored. Furthermore, horizontal violence among nurses is prevalent (7). As a typical form of negative workplace behavior (NWB) (8), it induces negative emotions (9), depletes nurses’ psychological resources, and impairs their job engagement (10). However, few studies have investigated the potential mechanism of horizontal violence in the relationship between transformational leadership style and job engagement within the nurse population. Therefore, this study intends to explore the mediating effect of horizontal violence among nurses on the relationship between head nurses’ transformational leadership style and nurses’ job engagement. The aim is to actively identify strategies to reduce horizontal violence among nurses and improve nurses’ job engagement, thereby ensuring patient safety and enhancing nursing quality.

### 1.1 Head nurse’s transformational leadership style and nurses’ job engagement

Leadership plays an increasingly important role in nursing management (11). Effective leadership can not only motivate nurses to adopt positive work behaviors but also improve nursing quality and promote the sound development of the nursing profession (12). Currently, nursing managers still face numerous challenges, with issues such as heavy workloads, low job engagement, and high turnover rates among nurses remaining prominent.

Based on the Social Exchange Theory (13), high-quality leader-member relationships (LMRs) are a key variable determining the level of members’ job engagement. This theory posits that effective LMRs should be characterized by mutual trust, mutual promotion, and mutual benefit, rather than being merely economic exchange relationships. Nursing practice requires continuous interaction with patients and team members, leading nurses to have a far greater demand for “socio-emotional resources” than for “economic resources.” As frontline managers in medical institutions who have direct contact with nurses, head nurses’ leadership styles directly affect the job engagement, cultural atmosphere, job satisfaction, and organizational cohesion of nurses in their departments (14). The transformational leadership style of head nurses focuses on motivating nurses to pursue higher goals by changing their attitudes, beliefs, values, and implementing humanistic management (15).

Studies have shown (16–18)that compared to the traditional transactional leadership style, nurses led by head nurses with a transformational leadership style exhibit higher self-efficacy and job engagement levels. Another study has indicated (19)that the transformational leadership style of head nurses can enhance nurses’ clinical abilities, especially in patient care, communication skills, management, and knowledge. Further research has noted (20) that transformational leadership emphasizes the concept of humanistic management more. Through personalized support (such as listening to nurses’ stress), it strengthens the cohesion of the nursing team, reduces horizontal violence among nurses, and thereby improves nurses’ job satisfaction and helps increase their retention rate. In light of the aforementioned theory and empirical findings, the following hypothesis is proposed:

*Hypothesis 1: There is a positive correlation between head nurses' transformational leadership style and nurses' job engagement.*

### 1.2 The mediating role of horizontal violence among nurses

Horizontal violence among nurses refers to hostile behaviors within the nursing profession (21). Its manifestations fall into two categories: overt and covert. Overt behaviors include belittling, slandering, and spreading rumors; covert behaviors involve concealing information, isolating and ignoring others, and obstructing promotion and learning opportunities (22, 23). The incidence of horizontal violence among nurses ranges from 7 to 83% (21), with significant variations across regions—partly influenced by local culture and environment (24). A systematic review indicates that nurses who have experienced horizontal violence are more likely to resign or change careers than those who have not (25). Additionally, horizontal violence reduces nurses’ willingness to collaborate, exacerbates job burnout, and leads to decreased job engagement (26).

Nurses work in high-intensity environments, so they have a greater need for a workplace characterized by mutual respect, polite communication, recognition of each other’s contributions, and civilized behavior. As a typical form of NWB (8), horizontal violence easily triggers internal conflicts within nursing teams when task assignments are unbalanced or resource allocation is unfair. In such negative contexts, nurses may exhibit behaviors like retaliation, avoidance, and complaining (27). The AET (28) posits that positive affective events can promote civil behaviors such as respect and collaboration, whereas negative affective events tend to trigger negative emotions like anxiety, depression, and tension (29). As a negative affective event, horizontal violence induces negative emotions in nurses, leading to reduced vitality, distracted attention, and decreased job engagement (26). The COR Theory further enriches this theoretical foundation: prolonged exposure to negative affective events continuously depletes nurses’ psychological resources, thereby reducing job satisfaction, increasing job insecurity, and ultimately resulting in decreased job engagement (30).

Currently, regarding the intrinsic relationships and interaction mechanisms among head nurses’ transformational leadership style, horizontal violence between nurses, and nurses’ job engagement, previous studies have mostly adopted a single theoretical perspective of the COR Theory (4). In contrast, this study integrates both the AET and COR Theory to construct a “Affective Event-Resource-Behavior” theoretical model. It aims to explore the potential interaction mechanisms between variables from two aspects: short-term triggering and long-term maintenance.

Based on these theories, we infer that the essence of how transformational leadership style influences job engagement may lie in shaping positive affective events (e.g., recognizing contributions, supporting innovation, providing care and assistance, and making fair decisions) while inhibiting negative affective events (i.e., horizontal violence among nurses). Accordingly, this study proposes the following hypotheses:

*Hypothesis 2: Horizontal violence among nurses is negatively correlated with both head nurses' transformational leadership style and nurses' job engagement levels.*

*Hypothesis 3: Horizontal violence among nurses plays a mediating role between head nurses' transformational leadership style and nurses' job engagement.*

## 2 Materials and methods

### 2.1 Study design and participants

From July to August 2024, a cross-sectional design was employed to randomly recruit 342 nurses from five tertiary grade A hospitals in Southwest China for survey analysis. A total of 14 questionnaires were excluded due to incomplete responses—specifically, at least 10% of the items were unanswered. After excluding 11 questionnaires with obvious patterns or unreasonable responses, 317 questionnaires were deemed eligible for final analysis, with an effective response rate of 92.69%.

The inclusion criteria for this study are as follows: registered nurses who are currently employed, have work experience of ≥1 year, have worked in the department where the current head nurse is located for ≥6 months, and have provided informed consent and are willing to cooperate voluntarily in completing this survey.

The exclusion criteria for this study are as follows: those who hold the position of head nurse, those who are not on duty due to external studies, training, sick leave, personal leave, maternity leave, etc., and retired, re-employed, or further-educated nurses.

The sample size was calculated using GPower 3.1.9.7 software (31). According to Cohen’s criteria (32), the effect size f^2^ was set to 0.15 (a medium effect size), the significance level (α) was set to 0.05, and the statistical power (1-β) was set to 80%. The total number of predictor variables in this study was 12. Based on these parameters, GPower calculated that the required sample size was 286 cases. To reduce the impact of multicollinearity and address missing data, a 10% increase in sample size was applied, resulting in an estimated required sample size of 315 cases. Finally, 342 nurses were invited to participate in the survey.

### 2.2 Ethical considerations

This study adheres to the ethical standards outlined in the Declaration of Helsinki and has been approved by the Ethics Committee of Deyang People’s Hospital (approval number: No. 2023–04-083-K01). Prior to the participation of all subjects, informed consent was obtained. The personal information of participants is private and confidential, and is handled anonymously.

### 2.3 Research tools

#### 2.3.1 General information survey form

This questionnaire is designed based on a comprehensive review of domestic and international literature, as well as expert consultations. It covers factors such as gender, age, educational level, marital status, professional title, years of work experience, number of night shifts per month, income status, and department affiliation.

#### 2.3.2 Transformational leadership style

The level of head nurses’ transformational leadership style is assessed using the Transformational Leadership Questionnaire (TLQ). The TLQ is a questionnaire designed to evaluate the perceived level of transformational leadership among employees. It was developed in 2005 by Li Chaoping and Shi Kan based on the Multifactor Leadership Questionnaire (MLQ) developed by Bass, and incorporates China’s national conditions and unique culture. It can be applied to corporate employees, teachers, workers, and medical staff, and is universally applicable and widely used (33, 34). It consists of four dimensions with 26 items, namely, eight items on moral exemplarity (items 1–8), six items on visionary inspiration (items 9–14), six items on personalized care (items 15–20), and six items on leadership charisma (items 21–26). The Likert 5-point scoring method is used, with scores ranging from 1 to 5, corresponding to the options of “Strongly Disagree,” “Somewhat Disagree,” “Unsure,” “Somewhat Agree,” and “Strongly Agree.” A higher score indicates a higher level of perceived transformational leadership behavior from the head nurse. The total score range is from 26 to 130, with a higher score indicating a higher level of transformational leadership. The Cronbach’s alpha coefficients for each dimension of the scale ranged from 0.840 to 0.920 (34). In this study, the Cronbach’s alpha coefficients for each dimension ranged from 0.805 to 0.890.

#### 2.3.3 Level of job engagement

The simplified version of the Utrecht Work Engagement Scale (UWES-9) was used to measure the nurses’ job engagement levels. Developed by Schaufeli et al. in 2006, this simplified version of the work engagement scale is designed to understand the level of employee engagement in their work (35). In 2012, Fong et al. (36)translated this scale into Chinese, thus forming the Chinese version of the work engagement scale. The UWES-9 comprises 3 dimensions with 9 items total: 3 for Vigor, 3 for Dedication, and 3 for Focus. All items use a 7-point Likert scale, with scores ranging from 0 to 6, corresponding to the options of “not at all,” “a few times per year,” “once a month,” “several times a month,” “once a week,” “several times a week,” and “every day.” The total score ranges from 0 to 54, where higher scores indicate greater job engagement among participants. Yang et al. (37)applied this scale to the nurse population and verified its good reliability, with a Cronbach’s α coefficient of 0.876. In this study, the Cronbach’s α coefficient was 0.955.

#### 2.3.4 Horizontal violence among nurses

The horizontal violence among nurses was measured using the “Nurse-to-Nurse Horizontal Violence Questionnaire” designed by Li Xiaoyan (38). The questionnaire consisted of two dimensions: explicit violence (8 items) and implicit violence (11 items). The frequency of horizontal violence experienced by the subjects over the past 3 months is rated on the Likert 5-point scale: 1 = “never experienced”; 2 = “yes, but rarely experienced”; 3 = “almost once a month”; 4 = “almost once a week”; and 5 = “almost once a day.” The total score ranged from 19 to 95, with a higher score indicating more severe horizontal violence. The Cronbach’s α coefficient of this questionnaire was 0.953 (39), indicating good reliability and validity. In this study, the Cronbach’s α coefficient was 0.952.

### 2.4 Data collection and quality control methods

The researchers obtained the consent of the nursing managers of the hospitals where the respondents worked, and distributed the questionnaires with their assistance. This survey was conducted using the WeChat-based “Questionnaire Star” platform. The questionnaire included explanations of the research purpose, significance, content, inclusion and exclusion criteria, survey time, etc., emphasizing the accuracy and reliability of the survey data. At the same time, the respondents were informed that their relevant information would be strictly confidential and used only for research purposes. Participants were informed that they could complete the survey voluntarily and anonymously. The first page and each section of the questionnaire included instructions for filling out the questionnaire. All items were required to be answered, and each IP address or WeChat account could only submit once, with a submission time limit set at 5 min. The collected questionnaires were carefully reviewed and entered by two researchers, and those with illogical answers, obvious errors, or regular patterns were excluded.

### 2.5 Statistical analyses

Analysis was conducted using SPSS 26.0. The general information of the subjects was described using frequency and percentage for categorical data, and mean and standard deviation for quantitative data. After conducting a normal distribution test on the data, Pearson correlation analysis was used to examine the correlation between head nurses’ transformational leadership style, nurses’ job engagement, and inter-nurse horizontal violence. Harman’s one-way ANOVA was used to detect common method bias. AMOS 26.0 software was used to construct a structural equation model with inter-nurse horizontal violence as the mediating variable. The mediating effect was tested using the bootstrap method, and the data were used for fitting and calibration, with a significance level of α = 0.05. The model fit indices were as follows: 1 < CMIN/DF < 5.000, RMSEA < 0.080, GFI, AGFI, NFI, CFI, IFI > 0.900, indicating good model fit.

## 3 Results

### 3.1 General information of the nurses under study and univariate analysis of job engagement among nurses with different characteristics

A total of 317 nurses were included in this study. The results showed that gender, age, marital status, and income had no significant impact on nurses’ job engagement, while educational background, professional title, years of work experience, and the number of night shifts per month had significant effects on nurses’ job engagement. Detailed data are presented in Table 1.

**Table 1:** Univariate analysis of nurses’ general data and job engagement with different characteristics.

Variables	*n* (%)	x̅ ± s	*t/F*	*p*
Gender				
Male	30 (5.36%)	34.93 ± 7.74	1.319	0.252
Female	287 (94.64%)	37.63 ± 12.61		
Age				
Aged 20–30	132 (41.64%)	36.09 ± 11.95	1.504	0.224
Aged 31–40	147 (46.37%)	38.62 ± 12.29		
Aged 41 and above	38 (11.99%)	37.03 ± 12.97		
Marital status				
Not married	64 (20.19%)	34.88 ± 13.24	1.708	0.183
Married	241 (76.03%)	37.97 ± 12.02		
Divorced	12 (3.79%)	38.83 ± 10.21		
Education level				
Junior college	63 (19.87%)	37.60 ± 10.19	5.688	0.004
Bachelor’s	236 (74.45%)	36.61 ± 12.76		
Master’s	18 (5.68%)	46.56 ± 7.84		
Professional title				
Junior nurse	155 (48.90%)	35.75 ± 12.20	4.719	0.010
Intermediate nurse	134 (42.27%)	38.07 ± 12.12		
Senior nurse	28 (8.83%)	43.07 ± 11.48		
Monthly Income(RMB)				
Up to 5,000	29 (9.15%)	34.38 ± 13.55	2.002	0.114
5,001–10,000	179 (56.47%)	38.50 ± 16.99		
10,001–15,000	63 (19.87%)	37.70 ± 11.02		
15,001 and above	46 (14.51%)	34.43 ± 14.50		
Years of service				
0–5 years	88 (27.76%)	33.24 ± 12.83	6.465	0.000
6–10 years	93 (29.34%)	39.24 ± 9.72		
11–20 years	91 (28.71%)	37.22 ± 13.68		
>20 years	45 (14.20%)	41.93 ± 10.42		
Number of night shifts per month				
0	124 (39.12%)	40.84 ± 11.33	9.964	0.000
1–5	84 (26.50%)	38.36 ± 11.46		
6–10	90 (28.39%)	33.23 ± 12.08		
>10	19 (5.99%)	30.05 ± 14.12		
Department				
Internal medicine	103 (32.49%)	36.84 ± 12.63	1.110	0.356
Surgery	99 (31.23%)	38.25 ± 10.82		
Outpatient and emergency department	22 (6.94%)	38.09 ± 12.93		
Intensive care unit	14 (4.42%)	30.21 ± 13.61		
Obstetrics, Gynecology and Pediatrics	37 (11.67%)	36.84 ± 14.66		
Operating room	18 (5.68%)	38.17 ± 10.33		
Other	24 (7.57%)	39.79 ± 11.76		

The score range of the Utrecht Work Engagement Scale is 0–54.

### 3.2 Scores for head nurses’ transformational leadership style, inter-nurse horizontal violence, and nurses’ job engagement

The total scores of head nurse’s transformational leadership style, inter-nurse horizontal violence, and nurses’ job engagement were (103.63 ± 14.60), (45.38 ± 9.72), and (37.38 ± 12.25), respectively. Detailed scores for each dimension and item are presented in Table 2.

**Table 2:** The scores of head nurses’ transformational leadership style、nurses’ job engagement and horizontal violence among nurses (n = 317).

Variables	Dimensions/items	Score range	Score situation
Head nurses’ transformational leadership style	26	26–130	103.63 ± 14.60
Moral Modeling	8	8–40	31.20 ± 4.69
Vision motivation	6	6–30	23.48 ± 3.81
Individualized Consideration	6	6–30	23.97 ± 4.45
Leadership Charisma	6	6–30	24.99 ± 3.54
Nurses’ job engagement	9	0–54	37.38 ± 12.25
Vigor	3	0–18	13.50 ± 3.99
Dedication	3	0–18	11.27 ± 4.30
Focus	3	0–18	12.60 ± 4.73
Horizontal violence among nurses	19	5–95	45.38 ± 9.72
Explicit violence	8	8–40	14.00 ± 4.77
Covert violence	11	11–55	31.38 ± 5.34

### 3.3 Analysis of the correlation between head nurses’ transformational leadership style, nurses’ job engagement, and interpersonal violence among nurses

A Pearson correlation analysis was conducted on the main variables and dimensions. The results indicated that there was a positive correlation between the transformational leadership style of head nurses and the level of nurses’ job engagement (*r* = 0.504, *p* < 0.001). There was a negative correlation between the transformational leadership style of head nurses and inter-nurse horizontal violence (*r* = −0.498, *p* < 0.001). Additionally, there was a negative correlation between inter-nurse horizontal violence and nurses’ job engagement (*r* = −0.505, *p* < 0.001). Detailed correlations among the variables and dimensions are presented in Table 3.

**Table 3:** The correlation between head nurses’ transformational leadership style, nurses’ job engagement, and horizontal violence among nurses (n = 317).

Variable	Head nurses’ transformational leadership style	Nurses’ job engagement	Horizontal violence among nurses
Head nurses’ transformational leadership style	1	-	-
Nurses’ job engagementt	0.504^**^	1	-
Horizontal violence among nurses	−0.498^**^	−0.505^**^	1
Moral model	0.881^**^	0.431^**^	−0.479^**^
Vision motivation	0.867^**^	0.450^**^	−0.418^**^
Individualized care	0.925^**^	0.436^**^	−0.416^**^
Leadership charisma	0.860^**^	0.474^**^	−0.446^**^
Vigor	0.501^**^	0.927^**^	−0.488^**^
Dedication	0.467^**^	0.960^**^	−0.532^**^
Focus	0.458^**^	0.935^**^	−0.412^**^
Explicit violence	−0.449^**^	−0.492^**^	0.957^**^
Hidden violence	−0.506^**^	−0.479^**^	0.966^**^

** Means *p* < 0.01.

### 3.4 Common method bias test

A common method bias test was conducted on all variables using Harman’s one-way ANOVA. The results indicated that the characteristic roots of nine factors were greater than 1, and the variance explained by the first factor was 36.148%, which was less than the critical value of 40%. This suggests that there is no significant common method bias in this study.

### 3.5 Analysis of the mediating effect of inter-nurse horizontal violence on head nurses’ transformational leadership style and nurses’ job engagement

**Figure 1:**
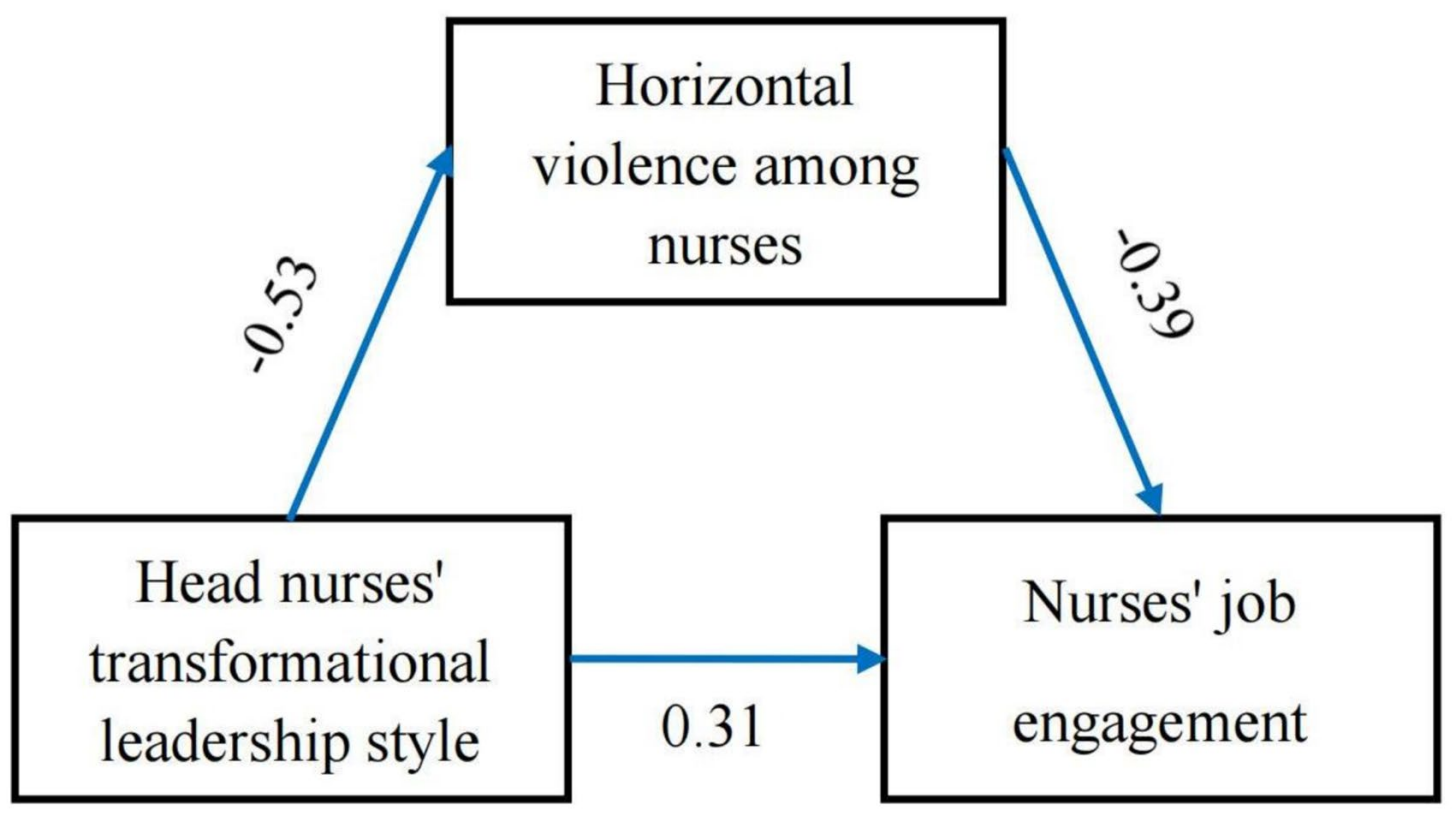
Mediating effect model of horizontal violence among nurses on head nurse leadership style and nurses’ job engagement.

Using Amos 26.0 software, a structural equation model was constructed with head nurses’ transformational leadership style as the independent variable, inter-nurse horizontal violence as the mediating variable, and nurses’ job engagement as the dependent variable. The model was fitted and revised using maximum likelihood method, and the final model is shown in Figure 1. The model fitting results are presented in Table 4. All parameters are within the ideal range, indicating that the model fits well.

**Table 4:** Structural equation model fitting index (standardized).

Project	Statistical tests	Suggested reference standards	Test results	Fit or not
Parsimony fit index	CMIN/DF	1–5	2.866	YES
Absolute fit indices	RMSEA	<0.08	0.077	YES
	GFI	>0.90	0.957	YES
	AGFI	>0.90	0.920	YES
Relative fit indices	NFI	>0.90	0.971	YES
	IFI	>0.90	0.981	YES
	CFI	>0.90	0.981	YES

CMIN/DF, Chi-square/degrees of freedom; RMSEA, Root Mean Square Error of Approximation; GFI, Goodness of Fit Index; AGFI, Adjusted Goodness of Fit Index; NFI, Normed Fit Index; IFI, Incremental Fit Index; CFI, Comparative Fit Index.

Using the Bootstrap method for 5,000 repeated samplings, with a confidence interval of 95% and excluding 0, we tested the mediating relationship between inter-nurse horizontal violence and the transformational leadership style of head nurses, as well as the level of nurses’ job engagement. The results indicated that the transformational leadership style of head nurses had a significant negative predictive effect on inter-nurse horizontal violence (β = −0.498, *p* < 0.001). Similarly, inter-nurse horizontal violence had a significant negative predictive effect on the level of nurses’ job engagement (β = −0.505, *p* < 0.001), supporting Hypothesis 2. Conversely, the transformational leadership style of head nurses had a significant positive predictive effect on the level of nurses’ job engagement (β = 0.504, *p* < 0.001), providing support for Hypothesis 1. Both the direct effect of the transformational leadership style of head nurses on nurses’ job engagement and the mediating effect of inter-nurse horizontal violence had 95% confidence intervals excluding 0, suggesting that inter-nurse horizontal violence partially mediates the relationship between the transformational leadership style of head nurses and nurses’ job engagement. The total effect of the transformational leadership style of head nurses on nurses’ job engagement was 0.517, while the mediating effect of inter-nurse horizontal violence was 0.207, accounting for 40.04% of the total effect, which supports Hypothesis 3. For details, please refer to Table 5.

**Table 5:** The mediating effect of horizontal violence among nurses on head nurses’ transformational leadership style and nurses’ job engagement.

Project	Standardized effect value	SE	95%CI	*p*	Relative effect value (%)
Direct effec	0.310	0.044	0.221–0.661	0.000	59.96%
Indirect effect	0.207	0.041	0.100–0.336	0.000	40.04%
Total effect	0.517	0.041	0.403–0.616	0.000	100.00%

## 4 Discussion

### 4.1 Analysis of the current status of head nurses’ transformational leadership style, horizontal violence among nurses, and nurses’ job engagement

The results of this study show that the score of head nurses’ transformational leadership style is (103.63 ± 14.60), which is higher than the scale’s median of 65, falling into the upper-middle level. Consistent with the research by Shen et al. (40), this finding indicates that nursing managers have gradually deepened their understanding of scientific management, continuously optimized head nurses’ leadership styles, and strengthened the construction of nursing teams (41). In this study, the score of the individualized care dimension is lower than that of previous relevant studies (40, 42). A possible reason is that when head nurses assign scientific research tasks beyond nurses’ capabilities, nurses bear dual pressures from clinical work and scientific research, making them more prone to job burnout and thus leading to a decrease in job engagement. This suggests that nursing managers should pay attention to nurses’ personal needs, abilities, and career expectations in their work, provide targeted training and guidance, and help nurses leverage their strengths and solve work-related problems.

In this study, the score of horizontal violence among nurses was (45.38 ± 9.72), which was below the scale’s median and lower than the result reported by Ke et al. (43). Analysis revealed that horizontal violence from senior nurses toward junior nurses was particularly prominent (39). However, this study did not include intern nurses or refresher nurses, resulting in a relatively low proportion of junior nurses in the sample. Another reason is that current hospitals attach great importance to humanistic management, with more transparent professional title promotion mechanisms and more reasonable salary systems—factors that have reduced the occurrence of horizontal violence among nurses to a certain extent.

Notably, the score of implicit violence in this study is above the scale’s median, which is consistent with the findings of Peng et al. (44). This indicates that implicit violence is still relatively common among nurses and is a precursor to explicit violence. Implicit violence is often overlooked, thus it is quite challenging for nursing managers to intervene. Some scholars have pointed out that the occurrence of inter-nurse horizontal violence is closely related to local culture (45). Guided by the Confucian values of “propriety” and “harmony as the most precious,” nurses tend to avoid overt conflicts when facing disputes and instead release negative emotions through implicit means. At the same time, nursing managers lack the ability to identify implicit violence, and nurses lack channels for communication and feedback—both factors contributing to the higher incidence of implicit violence.

In this study, the score of nurses’ job engagement was (37.38 ± 12.25), falling into the upper-middle level. This result is consistent with the findings of Shen et al. (46). Among all dimensions, the score for the Dedication was the lowest, while the scores for the Vigor and Focus were relatively higher. The main reason is that nurses must maintain a self-disciplined and rigorous attitude at work: they need to take each treatment procedure seriously and stay focused on work to better complete nursing tasks. Thus, the scores for Focus and Vigor are relatively high.

This study found that nurses had higher job engagement when they had fewer night shifts per month, longer work experience, or higher professional titles. A possible explanation is that nurses with longer work experience are more proficient in daily work. They also have wider interpersonal networks, access to more departmental resources, fewer night shifts, and a more regular lifestyle—all of which contribute to their higher job engagement. In addition, nurses with a master’s degree showed significantly higher job engagement than those with other educational backgrounds. This may be because individuals with a master’s degree were usually more skilled in learning, more diligent at work, and more willing to take the initiative to demonstrate their capabilities.

### 4.2 The correlation between head nurse’s transformational leadership style, nurses’ job engagement, and horizontal violence among nurses

This study shows that there is a positive correlation between head nurses’ transformational leadership style and nurses’ job engagement. Among its dimensions, the leadership charm dimension exhibits the strongest correlation with job engagement. This suggests that nurses led by head nurses with higher leadership charm demonstrate higher levels of job engagement, a result consistent with the study by Wang et al. (47). Based on the Social Exchange Theory (13), head nurses with a transformational leadership style are willing to change nurses’ attitudes, beliefs, and values through humanistic management, motivating nurses to pursue higher goals (48). Meanwhile, head nurses can give full play to their own charm and individualized care to create a positive working atmosphere for nursing practice. This can fully mobilize nurses’ internal motivation, stimulate strong work enthusiasm, and thereby enhance the level of nurses’ job engagement. This finding further suggests that head nurses themselves should also continue to learn and enrich their knowledge, improving their abilities in fair decision-making, communication, and handling negative emotional incidents. Additionally, head nurses should pay attention to the growth of individual nurses and nursing teams, providing individualized care for different nurses.

This study confirms a significant negative correlation between horizontal violence among nurses and job engagement. This conclusion not only aligns with existing research—for example, Reference (49) points out that horizontal violence increases nurses’ turnover rate, and Reference (26) finds that it exacerbates job burnout by inducing physical and mental harm such as insomnia, anxiety, and depression—but also analyzes its core theoretical mechanism by integrating the COR Theory and AET: “horizontal violence incidents lead to resource depletion, which in turn results in decreased job engagement. “This finding indicates that management strategies need to be optimized from the perspective of “proactive resource protection.” It is essential not only to focus on intervention after violence occurs but also to reduce horizontal violence at its source in a timely manner and identify potential horizontal violence (especially implicit violence) promptly. This will enable a transition from “passive response to problems” to “proactive resource maintenance,” and ultimately enhance nurses’ job engagement through resource protection, providing a fundamental guarantee for nursing quality and patient safety.

### 4.3 The mediating effect of horizontal violence among nurses on head nurses’ transformational leadership style and nurses’ job engagement

The survey results indicate that horizontal violence among nurses plays a partial mediating role in the relationship between head nurses’ transformational leadership style and nurses’ job engagement, with the mediating effect accounting for 40.04% of the total effect. This suggests that head nurses’ transformational leadership style not only directly influences the level of nurses’ job engagement but also exerts an indirect impact through horizontal violence among nurses.

From the integrated perspective of the AET and COR Theory, AET focuses on the short-term transmission path of “affective events to individual behaviors,” (50)explaining how horizontal violence leads to a decline in job engagement in the short term. Specifically, the characteristics of transformational leadership—such as supporting innovation, making fair decisions, recognizing contributions, fostering a positive organizational culture, and providing humanistic care to employees (5, 19, 20)—can be regarded as positive affective events, which promote an increase in nurses’ job engagement. In contrast, horizontal violence among nurses is a negative affective event, which leads to a decrease in nurses’ job engagement. The COR Theory, on the other hand, emphasizes the long-term evolutionary process of “resource depletion-psychological imbalance-decline in job engagement,” (51)providing theoretical support for why horizontal violence can continuously inhibit job engagement. Specifically, when nurses experience horizontal violence, their psychological resources are continuously depleted, and insufficient work resources further reduce their job engagement. However, head nurses with a transformational leadership style can enhance nurses’ job engagement by creating more positive affective events and reducing the impact of negative affective events, thereby preventing the depletion of nurses’ psychological resources and promoting their recovery.

## 5 Conclusion

The results of this study indicate that there is still room for improvement in the job engagement of Chinese nurses, and the issue of horizontal violence among nurses remains prominent. Head nurses’ transformational leadership style can significantly and positively predict the level of nurses’ job engagement; meanwhile, horizontal violence among nurses plays a partial mediating role in the relationship between head nurses’ transformational leadership style and nurses’ job engagement. This study integrated the AET and COR Theory to construct an “Affective Event-Resource-Behavior” theoretical model, and the research results validated this model. It explains how head nurses’ transformational leadership improves nurses’ job engagement by reducing horizontal violence, which provides a new perspective for the innovation of theoretical application in the field of nursing management.

## 6 Practical implications

It is recommended that nursing administrators implement interventions from the dual dimensions of affective event regulation and resource depletion interruption to improve nurses’ job engagement and sense of well-being, thereby enhancing nursing quality. The specific plans are as follows:

The nursing department, in collaboration with the psychosomatic medicine department, shall develop a classification list of horizontal violence among nurses, which shall include both implicit and explicit violence lists and be presented in a combination of text and comics.

An anonymous horizontal violence incident reporting system shall be integrated into the hospital intranet, covering violence type, occurrence frequency, and involved personnel, to provide a feedback channel for those who have experienced violence.

A database of horizontal violence cases shall be established by collecting real in-hospital cases. Nursing administrators can conduct scenario-based role-playing training to recreate real situations, and all participants shall sign an anti-violence agreement upon completion of the training.

Head nurses shall strengthen the construction of departmental team culture, develop personalized care manuals, increase communication with nurses, promptly understand nurses’work-related psychological status, and document such information.

## 7 Study limitations

First, due to constraints of objective conditions such as human and material resources, this study only surveyed nurses from selected hospitals in Southwest China. This has led to certain limitations in the representativeness of the sample, and the research findings may be influenced by factors such as regional characteristics and economic development levels. Future studies could verify the conclusions through surveys conducted across different regions, demographic groups, and with larger sample sizes. Second, this study adopted a cross-sectional design, collecting concurrent data at a single time point. Consequently, it is unable to clarify the temporal sequence and causal direction among variables. In the future, a longitudinal follow-up design could be employed to rule out the impacts of reverse causality and confounding variables. Third, all core variables in this study were collected through nurse self-reported questionnaires. Although measures such as “anonymous completion” and “emphasis on data confidentiality” were implemented to reduce bias, the inherent limitations of self-report methods cannot be completely avoided, which may affect the accuracy of the results. Future research could integrate multi-source data, such as colleague ratings and head nurse evaluations, to cross-validate data authenticity and mitigate self-report bias. Finally, due to limitations imposed by regional cultural differences, caution is required when generalizing the research conclusions to nursing teams in other cultural contexts. Future studies could conduct cross-regional and cross-cultural comparative research to expand the applicability of the findings.

## Data Availability

The datasets presented in this study can be found in online repositories. The names of the repository/repositories and accession number(s) can be found at: the repository is figshare. Data link doi: 10.6084/m9.figshare.28830959.

## References

[1] Zhai Y Cai S Chen X Zhao W Yu J Zhang Y. The relationships between organizational culture and thriving at work among nurses: the mediating role of affective commitment and work engagement. J Adv Nurs. (2023) 79:194–204. doi: 10.1111/jan.15443, PMID: 36104977

[2] Liu Y Zhang F Guan C Song B Zhang H Fu M. Patient satisfaction with humanistic nursing in Chinese secondary and tertiary public hospitals: a cross-sectional survey. Front Public Health. (2023) 11:1163351. doi: 10.3389/fpubh.2023.1163351, PMID: 37711237 PMC10498541

[3] Wang X Liu L Zou F Hao J Wu H. Associations of occupational stressors, perceived organizational support, and psychological capital with work engagement among Chinese female nurses. Biomed Res Int. (2017) 2017:1–11. doi: 10.1155/2017/5284628, PMID: 28168198 PMC5266809

[4] Huang Q Wang L Huang H Tang H Liu J Chen C. Transformational leadership, psychological empowerment, work engagement and intensive care nurses' job performance: a cross-sectional study using structural equation modeling. BMC Nurs. (2025) 24:1025. doi: 10.1186/s12912-025-03685-7, PMID: 40764543 PMC12323184

[5] Agaoglu FO Bas M Tarsuslu S Ekinci LO. Serial mediating role of transformational leadership and perception of artificial intelligence use in the effect of employee happiness on innovative work behaviour in nurses. BMC Nurs. (2025) 24:137. doi: 10.1186/s12912-025-02776-9, PMID: 39915749 PMC11800594

[6] Alanazi NH Alshamlani Y Baker OG. The association between nurse managers' transformational leadership and quality of patient care: a systematic review. Int Nurs Rev. (2023) 70:175–84. doi: 10.1111/inr.12819, PMID: 36583960

[7] Blackstock S Salami B Cummings GG. Organisational Antecedents, Policy and Horizontal Violence among Nurses: An Integrative Review. J Nurs Manag (2018) 26:972–91. Epub 20180831. doi: 10.1111/jonm.12623 30171643

[8] Verschuren CM Tims M de Lange AH. A systematic review of negative work behavior: toward an integrated definition. Front Psychol. (2021) 12:726973. doi: 10.3389/fpsyg.2021.726973, PMID: 34777108 PMC8578924

[9] Pagnucci N Ottonello G Capponi D Catania G Zanini M Aleo G. Predictors of events of violence or aggression against nurses in the workplace: a scoping review. J Nurs Manag. (2022) 30:1724–49. doi: 10.1111/jonm.13635, PMID: 35420236 PMC9796891

[10] Fujii C Iwasa Y. Implications of nursing peer violence on patient safety: An integrative review. Int Nurs Rev. (2025) 72:e70042. doi: 10.1111/inr.70042, PMID: 40607599 PMC12224223

[11] Garcia-Sierra R Fernandez-Castro J. Relationships between leadership, structural empowerment, and engagement in nurses. J Adv Nurs. (2018) 74:2809–19. doi: 10.1111/jan.13805, PMID: 30019477

[12] Cummings GG Lee S Tate K Penconek T Micaroni SPM Paananen T. The essentials of nursing leadership: a systematic review of factors and educational interventions influencing nursing leadership. Int J Nurs Stud. (2021) 115:103842. doi: 10.1016/j.ijnurstu.2020.103842, PMID: 33383271

[13] Zhang F Huang L Fei Y Peng X Liu Y Zhang N. Impact of caring leadership on nurses' work engagement: examining the chain mediating effect of calling and affective organization commitment. BMC Nurs. (2024) 23:716. doi: 10.1186/s12912-024-02388-9, PMID: 39370507 PMC11456229

[14] Zaghini F Fiorini J Piredda M Fida R Sili A. The relationship between nurse managers' leadership style and patients' perception of the quality of the care provided by nurses: cross sectional survey. Int J Nurs Stud. (2020) 101:103446. doi: 10.1016/j.ijnurstu.2019.103446, PMID: 31670220

[15] Manning J. The influence of nurse manager leadership style on staff nurse work engagement. J Nurs Adm. (2016) 46:438–43. doi: 10.1097/NNA.0000000000000372, PMID: 27496584

[16] Al-Rjoub S Alsharawneh A Alhawajreh MJ Othman EH. Exploring the impact of transformational and transactional style of leadership on nursing care performance and patient outcomes. J Healthc Leadersh. (2024) 16:557–68. doi: 10.2147/JHL.S496266 39742286 PMC11687278

[17] Richards A. Exploring the benefits and limitations of transactional leadership in healthcare. Nurs Stand. (2020) 35:46–50. doi: 10.7748/ns.2020.e11593, PMID: 33089675

[18] Alluhaybi A Usher K Durkin J Wilson A. Clinical nurse managers' leadership styles and staff nurses' work engagement in Saudi Arabia: a cross-sectional study. PLoS One. (2024) 19:e0296082. doi: 10.1371/journal.pone.0296082, PMID: 38452098 PMC10919612

[19] Lin YW Ni CF Hsu SF Tsay SL Tung HH. Effects of length of employment and head nurse leadership style on the clinical competency of staff nurses in Taiwan. J Nurs Res. (2024) 32:e331. doi: 10.1097/jnr.0000000000000617, PMID: 38814996

[20] Kaiser JA. The relationship between leadership style and nurse-to-nurse incivility: turning the Lens inward. J Nurs Manag. (2017) 25:110–8. doi: 10.1111/jonm.12447, PMID: 27896878

[21] Zhang Y Cai J Yin R Qin S Wang H Shi X. Prevalence of lateral violence in nurse workplace: a systematic review and Meta-analysis. BMJ Open. (2022) 12:e054014. doi: 10.1136/bmjopen-2021-054014, PMID: 35351708 PMC8966576

[22] Huang S Kong G Li Q Lilenga HS Zhai J. Status of horizontal violence, level of psychological empowerment, and their correlation among obstetric nurses: a cross-sectional survey. Birth. (2024) 52:252–60. doi: 10.1111/birt.12879, PMID: 39462967

[23] Pina D Vidal-Alves M Puente-Lopez E Luna-Maldonado A Luna Ruiz-Cabello A Magalhaes T. Profiles of lateral violence in nursing personnel of the Spanish public health system. PLoS One. (2022) 17:e0268636. doi: 10.1371/journal.pone.0268636, PMID: 35622880 PMC9140284

[24] Travaini GV Flutti E Sottocornola M Tambone V Blandino A Di Palma G. Evidence of Horizontal Violence in Healthcare Settings: A Narrative Review. Nurs Rep (2024) 14:1647–60. doi: 10.3390/nursrep14030123 39051359 PMC11270318

[25] Zhang Y Yin R Lu J Cai J Wang H Shi X. Association between horizontal violence and turnover intention in nurses: a systematic review and Meta-analysis. Front Public Health. (2022) 10:964629. doi: 10.3389/fpubh.2022.964629, PMID: 36276344 PMC9583538

[26] Liu M Wang Z Yan Z Wei H Wang Y Wang Y. Status quo and influencing factors of posttraumatic growth of nurses exposed to nurse-to-nurse horizontal violence: a cross-sectional multicenter study. BMC Nurs. (2024) 23:937. doi: 10.1186/s12912-024-02609-1, PMID: 39707314 PMC11660454

[27] Zou J Yang Y Chen L Bi Y Li N Luo Q. Effects of negative workplace behavior on job insecurity and turnover intention in healthcare workers: roles of psychological resilience. Front Public Health. (2025) 13:1493964. doi: 10.3389/fpubh.2025.1493964, PMID: 40492010 PMC12146628

[28] Itzkovich Y Heilbrunn S Dolev N. Drivers of intrapreneurship: an affective events theory viewpoint. Pers Rev. (2021) 51:1449–70. doi: 10.1108/PR-09-2019-0483

[29] Layne DM Nemeth LS Mueller M Wallston KA. The negative behaviors in healthcare survey: instrument development and validation. J Nurs Meas. (2019) 27:221–33. doi: 10.1891/1061-3749.27.2.221, PMID: 31511406

[30] Prapanjaroensin A Patrician PA Vance DE. Conservation of resources theory in nurse burnout and patient safety. J Adv Nurs. (2017) 73:2558–65. doi: 10.1111/jan.13348, PMID: 28543427

[31] Faul F Erdfelder E Lang AG Buchner A. G*power 3: a flexible statistical power analysis program for the social, behavioral, and biomedical sciences. Behav Res Methods. (2007) 39:175–91. doi: 10.3758/BF03193146, PMID: 17695343

[32] Faul F Erdfelder E Buchner A Lang AG. Statistical power analyses using G*power 3.1: tests for correlation and regression analyses. Behav Res Methods. (2009) 41:1149–60. doi: 10.3758/BRM.41.4.1149, PMID: 19897823

[33] Al-Thawabiya A Singh K Al-Lenjawi BA Alomari A. Leadership styles and transformational leadership skills among nurse leaders in Qatar, a cross-sectional study. Nurs Open. (2023) 10:3440–6. doi: 10.1002/nop2.1636, PMID: 36760040 PMC10170951

[34] Chu H Qiang B Zhou J Qiu X Yang X Qiao Z. The impact of transformational leadership on physicians' performance in China: a cross-level mediation model. Front Psychol. (2021) 12:586475. doi: 10.3389/fpsyg.2021.586475, PMID: 33790823 PMC8006430

[35] Kulikowski K. Do we all agree on how to measure work engagement? Factorial validity of Utrecht work engagement scale as a standard measurement tool - a literature review. Int J Occup Med Environ Health. (2017) 30:161–75. doi: 10.13075/ijomeh.1896.00947, PMID: 28366949

[36] Fong TC Ng SM. Measuring engagement at work: validation of the Chinese version of the Utrecht work engagement scale. Int J Behav Med. (2012) 19:391–7. doi: 10.1007/s12529-011-9173-6, PMID: 21681564 PMC3422451

[37] Yang G Wei H Wan L Dong H Liang X He Y. Curvilinear relationship between burnout and work engagement among staff in Community Services for the Elderly: a correlation study. Front Public Health. (2022) 10:939649. doi: 10.3389/fpubh.2022.939649, PMID: 35937238 PMC9354742

[38] Li X Li W An L. The current status of research on the causes and countermeasures of horizontal violence among nurses. Chin J Nurs. (2009) 44:886–8. doi: 10.3761/j.issn.0254-1769.2009.10.005

[39] Krut BA Laing CM Moules NJ Estefan A. The impact of horizontal violence on the individual nurse: a qualitative research study. Nurse Educ Pract. (2021) 54:103079. doi: 10.1016/j.nepr.2021.103079, PMID: 34089972

[40] Shen Y Zhang Y. Mediating effect of cognitive errors on perceived transformational leadership of head nurses and innovative behavior of nurses. J Nurs. (2019) 26:51–4. doi: 10.16460/j.issn1008-9969.2019.21.051

[41] Asiri SA Rohrer WW Al-Surimi K Da'ar OO Ahmed A. The association of leadership styles and empowerment with nurses' organizational commitment in an acute health care setting: a cross-sectional study. BMC Nurs. (2016) 15:38. doi: 10.1186/s12912-016-0161-7 27293380 PMC4901399

[42] Yuan Z Liu H. Influence of head nurses' transformational leadership behaviors on ICU nurses' innovative efficacy. Chin Nurs Manag. (2017) 17:1039–42. doi: 10.3969/j.issn.1672-1756.2017.08.008

[43] Ke Y Wu S Zhu H Yan L Jin Q. Influence of lateral violence amongst nurses on the solidarity degree of clinical nurses. CJGP. (2022) 20:161–4. doi: 10.16766/j.cnki.issn.1674-4152.002302

[44] Peng X Gan Y Zeng Q Xiong L Zhang F Xiong H. Nurse-to-nurse horizontal violence in Chinese hospitals and the protective role of head nurse's caring and Nurses' Group behaviour on it: a cross-sectional study. J Nurs Manag. (2022) 30:1590–9. doi: 10.1111/jonm.13498, PMID: 34699090 PMC9787125

[45] Bambi S Foa C De Felippis C Lucchini A Guazzini A Rasero L. Workplace incivility, lateral violence and bullying among nurses. A review about their prevalence and related factors. Acta Biomed. (2018) 89:51–79. doi: 10.23750/abm.v89i6-S.7461, PMID: 30038204 PMC6357596

[46] Shen X Shen T Chen Y Wang Y He X Lv X. The associations between benevolent leadership, affective commitment, work engagement and helping behavior of nurses: a cross-sectional study. BMC Nurs. (2023) 22:407. doi: 10.1186/s12912-023-01581-6, PMID: 37904189 PMC10614312

[47] Wang Y Zheng X Dong X Zhou W Wan Q Shang S. A structural equation modeling study on the impact of transformational leadership style and structural empowerment on doctor-nurse collaboration. Chin Nurs Manag. (2022) 22:77–82. doi: 10.3936/j.issn.1671-315x.2022.02.001

[48] Gashaye M Tilahun D Belay A Bereka B. Perceived Utilization of Leadership Styles among Nurses. Risk Manag Healthc Policy (2023) 16:215–24. Epub 20230211. doi: 10.2147/RMHP.S388966 36819844 PMC9930582

[49] Ebrahimi H Hassankhani H Negarandeh R Jeffrey C Azizi A. Violence against new graduated nurses in clinical settings: a qualitative study. Nurs Ethics. (2017) 24:704–15. doi: 10.1177/0969733015624486, PMID: 26811399

[50] Bleidorn W Hopwood CJ Lucas RE. Life events and personality trait change. J Pers. (2018) 86:83–96. doi: 10.1111/jopy.12286, PMID: 27716921

[51] Griep Y Bankins S Vander Elst T De Witte H. How psychological contract breach affects long-term mental and physical health: the longitudinal role of effort-reward imbalance. Appl Psychol Health Well Being. (2021) 13:263–81. doi: 10.1111/aphw.12246, PMID: 33492770 PMC8248376

